# Anterior Segment Optical Coherence Tomography Angiography Following Trabecular Bypass Minimally Invasive Glaucoma Surgery

**DOI:** 10.3389/fmed.2022.830678

**Published:** 2022-03-07

**Authors:** Jinyuan Gan, Chelvin C. A. Sng, Mengyuan Ke, Chew Shi Chieh, Bingyao Tan, Leopold Schmetterer, Marcus Ang

**Affiliations:** ^1^Duke-NUS Graduate Medical School, Singapore, Singapore; ^2^Singapore National Eye Centre, Singhealth, Singapore Eye Research Institute, Singapore, Singapore; ^3^Department of Ophthalmology, National University Hospital, Singapore, Singapore; ^4^SERI-NTU Advanced Ocular Engineering (STANCE), Singapore, Singapore; ^5^School of Chemical and Biomedical Engineering, Nanyang Technological University, Singapore, Singapore; ^6^Department of Clinical Pharmacology, Medical University of Vienna, Vienna, Austria; ^7^Center for Medical Physics and Biomedical Engineering, Medical University of Vienna, Vienna, Austria; ^8^Institute of Molecular and Clinical Ophthalmology, Basel, Switzerland

**Keywords:** glaucoma, imaging, intraocular pressure, sclera, cornea, episclera

## Abstract

**Objective:**

To assess anterior segment optical coherence tomography angiography (AS-OCTA) imaging of the episcleral vessels before and after trabecular bypass minimally invasive glaucoma surgery (MIGS).

**Design:**

A prospective, clinical, single-centre, single-arm pilot feasibility study conducted at National University Hospital, Singapore.

**Subjects:**

Patients with primary glaucomatous optic neuropathy undergoing Hydrus Microstent (Ivantis Inc., Irvine, CA, USA) implantation, who require at least one intra-ocular pressure-lowering medication. One or two eyes per patient may be enrolled.

**Methods:**

We performed AS-OCTA (Nidek RS-3000 Advance 2, Gamagori, Japan) pre- and up to 6 months post-MIGS implantation using a standard protocol in all cornealimbal quadrants, to derive episcleral vessel densities (VD) using a previously described technique.

**Main Outcome Measures:**

Episcleral VD pre- and post-surgery, in sectors with and without the implant.

**Results:**

We obtained serial AS-OCTA images in 25 eyes undergoing MIGS implantation (23 subjects, mean age 70.3 ± 1.5, 61% female) with mean preoperative intraocular pressure (IOP) of 15.5 mmHg ± 4.0. We observed reductions in postoperative episcleral VD compared to preoperative VD at month 1 (mean difference −3.2, *p* = 0.001), month 3 (mean difference −2.94, *p* = 0.004) and month 6 (mean difference −2.19, *p* = 0.039) in sectors with implants (overall 6 month follow-up, *p* = 0.011). No significant changes were detected in episcleral VD in the sectors without implants (*p* = 0.910).

**Conclusion:**

In our pilot study, AS-OCTA was able to detect changes in the episcleral VD following trabecular bypass MIGS, which may be a useful modality to evaluate surgical outcomes if validated in future studies.

## Introduction

Glaucoma is one of the leading causes of blindness worldwide (1). Increased intraocular pressure (IOP) is the main risk factor for glaucoma, and the mainstay of glaucoma treatment involves lowering of IOP (2, 3). The most common treatment involves the use of topical medications – however, these may be associated with adverse effects and poor compliance (4). Meanwhile conventional glaucoma surgeries such as trabeculectomy may effectively lower IOP, but can be associated with sight-threatening complications (5). To address these limitations, minimally invasive glaucoma surgery (MIGS) has gained popularity in recent years.

Currently, MIGS include a heterogeneous group of IOP-lowering devices and procedures that are generally less invasive and have a faster recovery time compared to traditional filtration surgery (6, 7). While MIGS is usually associated with a good safety profile, clinical results suggest variable efficacy in IOP reduction (8, 9). Both iStent (Glaukos, San Clemente, CA, USA) and Hydrus Microstent (Ivantis Inc., Irvine, CA, USA) are ab interno trabecular bypass products that increase aqueous outflow, with the latter scaffolding and dilating the Schlemm's canal as well. In a head-to-head study comparing Hydrus to iStent inject, the COMPARE study found Hydrus to have a greater rate of surgical success compared to iStent, with fewer subjects needing repeat glaucoma surgeries or medications (10).

When evaluating trabecular bypass MIGS devices, imaging the aqueous outflow tracts may be useful in understanding its efficacy. Aqueous angiography is a functional imaging technique utilising an ab interno approach with fluorescein or indocyanine green (ICG) as tracers, demonstrated in enucleated animal eyes (11, 12) and *in vivo* animal studies (13). However, aqueous angiography has limited clinical application as it is an invasive procedure that requires intraocular injection of dye, and is associated with potential complications such as infection and anaphylaxis (14). Recently, optical coherence tomography angiography (OCTA) has emerged as a non-invasive, rapid imaging technique that may be used to delineate vasculature in the anterior segment (15). While the role of anterior segment OCTA (AS-OCTA) has been described for episcleral, scleral and limbal vasculature (16–21), it has not been described specifically for the episcleral venous plexus in relation to MIGS to date (22). Thus, we conducted this pilot feasibility study to evaluate the role of AS-OCTA imaging following Hydrus Microstent implantation, to examine the potential effect of this trabecular bypass MIGS implant on episcleral vessel density.

## Materials and Methods

This was a prospective single-centre case series of consecutive patients who underwent combined phacoemulsification with Hydrus Microstent implantation at the National University Hospital between May 2019 to Mar 2020. Approval was obtained from the National Healthcare Group Domain Specific Review Board (2016/00125) and the study was conducted in accordance with the tenets of the Declaration of Helsinki. Written informed consent was obtained from all patients prior to surgery.

### Study Subjects

We included phakic subjects with primary glaucomatous optic neuropathy, as defined by Foster et al. (23), who required at least one intraocular-pressure lowering medication in this study. Exclusion criteria included advanced primary angle-closure glaucoma (PACG) (24) (as defined by cup-disc ratio ≥0.9 and/or a visual field defect within the central 10° of fixation), >180° of peripheral anterior synechiae, peripheral anterior synechiae in the target quadrant of Hydrus Microstent implantation, prior incisional glaucoma surgery, secondary glaucoma (including uveitic, neovascular, traumatic glaucoma, or glaucoma secondary to raised episcleral venous pressure) and any orbital, corneal, retinal or choroidal disease which may interfere with cataract extraction or Hydrus Microstent implantation.

### Study Measures

Complete ophthalmic examination by a fellowship-trained glaucoma specialist (C. A. Sng) was performed pre-operatively and on day 1, week 1, and months 1, 3, and 6. This included the best corrected Snellen visual acuity (BCVA), IOP measurement with Goldmann applanation tonometry, and a detailed slit lamp examination of the anterior and posterior segments. Humphrey perimetry (Swedish Interative Threshold Algorithm Standard 24-2 algorithm, Humphrey Visual Field, HVF Analyzer II, Carl Zeiss Meditec, Inc., Dublin, California, USA) was performed pre-operatively and 6 months post-operatively, and the mean deviation (MD) was recorded.

### Surgical Technique

All surgeries were performed under topical anaesthesia or peribulbar block. Phacoemulsification and intraocular lens implantation were performed via a clear corneal incision. To implant the Hydrus Microstent, the surgical microscope was tilted 30° towards the patient and the patient's head was tilted 45° nasally or inferiorly to allow direct visualisation of the angle structures with an intra-operative gonioscopy lens (Ocular Hill Open Access Surgical Gonioscopy [Left-Hand], Ocular Instruments, Bellevue, WA). An ophthalmic viscosurgical device was used to maintain the anterior chamber and widen the anterior chamber angle after phacoemulsification. The Hydrus Microstent was passed into the anterior chamber through a separate clear corneal incision (about 90 to 120° from the target site of Hydrus Microstent implantation) into the anterior chamber. The trabecular meshwork was incised with the tip of the device injector cannula and the Hydrus Microstent was inserted into the Schlemm's canal in the nasal or inferior quadrant over a span of approximately 90°. The targeted quadrants were reported to contain greater aqueous humour outflow (AHO), and selection of quadrants was based on surgical accessibility through a clear corneal temporary incision, and surgical technique of Hydrus Microstent implantation (14, 25). After visual confirmation of correct device positioning with the Schlemm's canal, the device injector was withdrawn and the ophthalmic viscosurgical device was removed. The corneal incisions were hydrated with a balanced salt solution. Vision blue (D.O.R.C. Dutch Ophthalmic Research Center [International] B.V., Zuidland, The Netherlands) was injected into the anterior chamber and the presence of the blue dye in the conjunctival vessels was noted and videoed for manual segmentation and comparison with OCTA vessels.

### Anterior Segment Imaging

**Figure 1:**
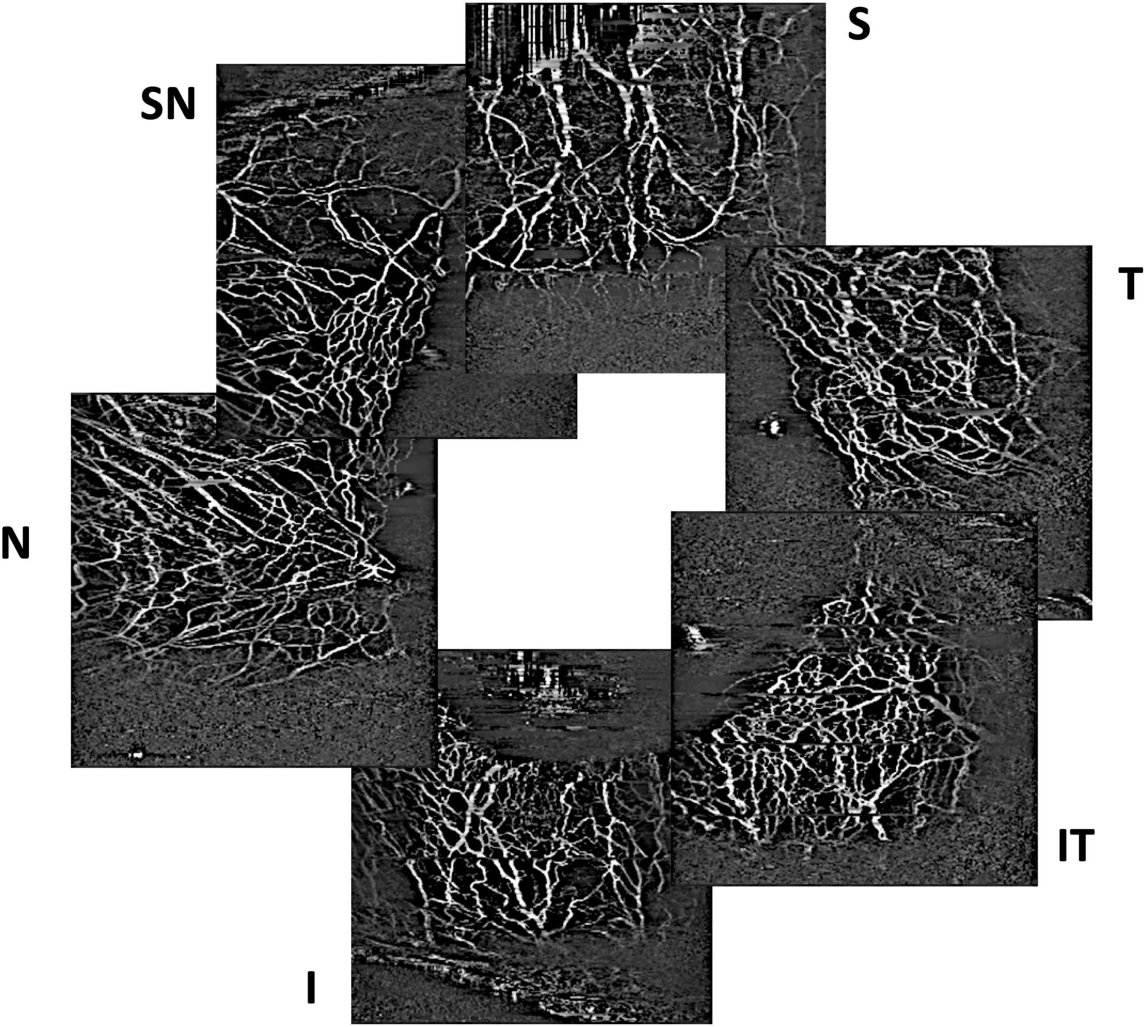
Example of optical coherence tomography angiography scans of episcleral vessels acquired in sectors with the trabecular bypass minimally invasive glaucoma surgical device (S, superior; SN, superior nasal; N, nasal) and control sectors without the implant (I, inferior; IT, inferior temporal; T, temporal).

Anterior segment imaging was performed pre-operatively and post-operatively at week 1, month 1, month 3, and month 6 using a digital slit-lamp camera (Topcon ATE-600, Nikon Corp) with a standard diffuse illumination (×10 magnification, flash power 4) for colour photography. Next, AS-OCTA of the episcleral vessels in all cornealimbal quadrants was conducted using a previously described scan protocol (26), using a spectral domain optical coherence tomography system (Nidek RS-3000 Advance 2, Gamagori, Japan) with a central wavelength of 880 nm, axial resolution of 7 um and transverse resolution of 15 um (anterior segment module). The eye tracker function was deactivated for imaging acquisition. The lens was moved close to the area of interest at the corneal surface before optimisation of the focal length and Z position to focus on the area of interest. The scan areas were divided into six sectors: Superior, superior nasal (right eye), nasal, inferior nasal, inferior, inferior temporal (left eye) and temporal directions i.e., six scans were acquired for each eye (Figure 1).

### AS-OCTA Image Processing

**Figure 2:**
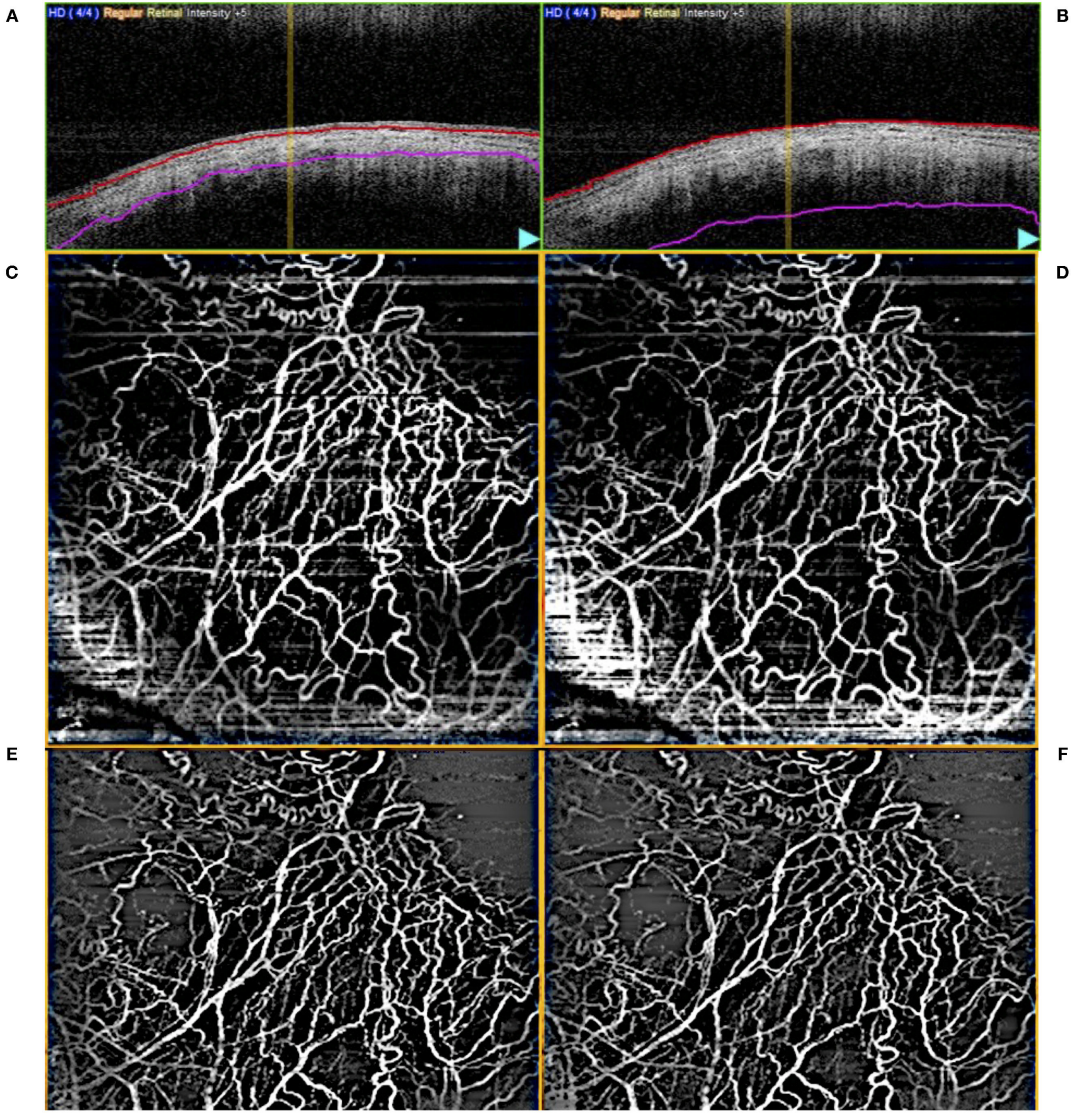
Example of **(A)** episcleral and **(B)** full layer (conjunctival, episcleral, scleral) segmented optical coherence tomography angiography (AS-OCTA) images. Unprocessed en face AS-OCTA images of the **(C)** episcleral and **(D)** full layer vasculature, respectively, and after removal of artefacts [**(E,F)**, respectively].

**Figure 3:**
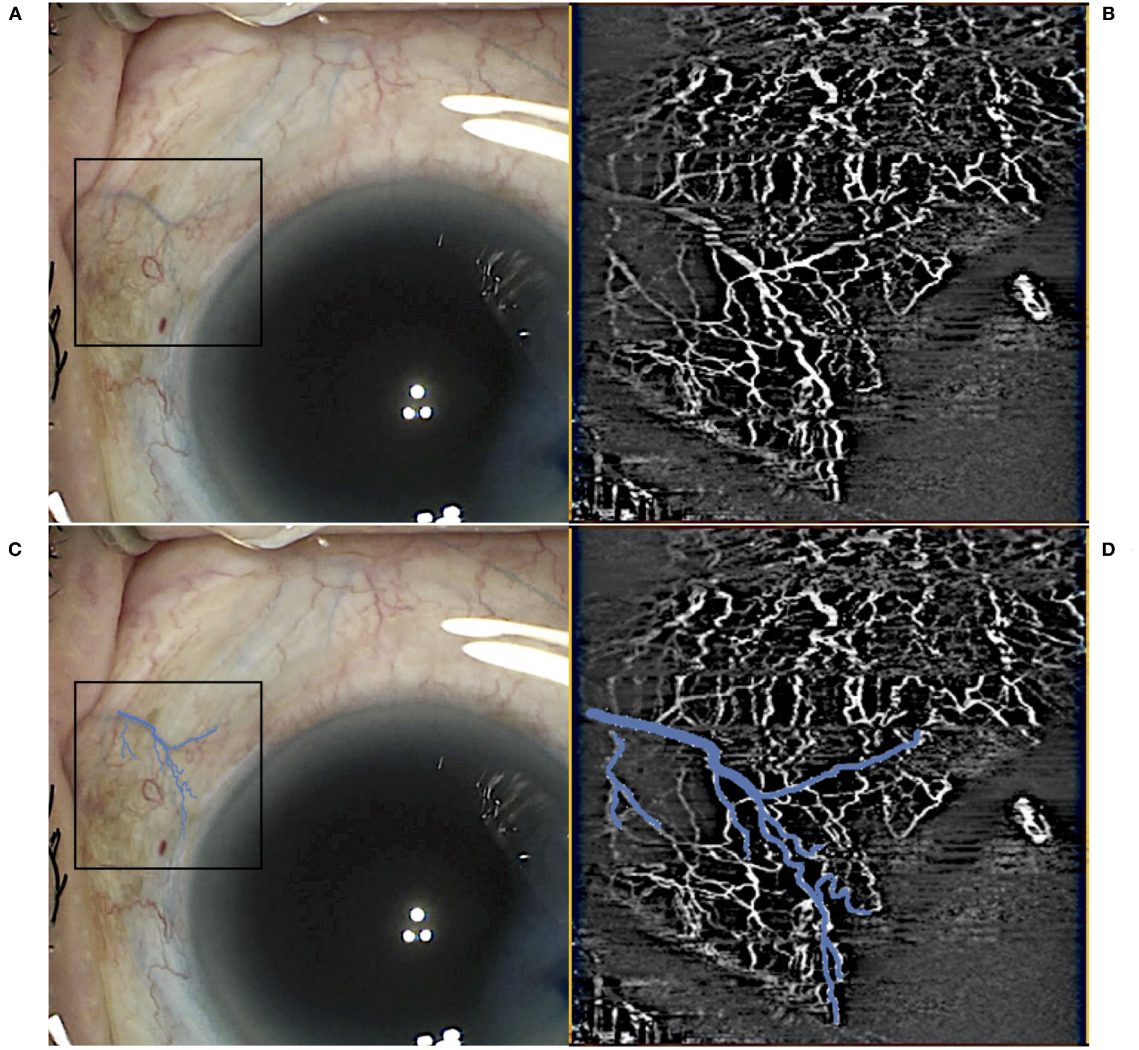
Example of intraoperative identification of episcleral vessels using trypan blue immediately postimplant **(A)** with the corresponding optical coherence tomography angiography (OCTA) scan in the sector **(B)**. Trypan blue vessels in the region of interest were highlighted **(C)** with an overlay on the intraoperative images **(D)** before vessel density calculations.

Scans were segmented manually to produce AS-OCTA enface images of (a) episcleral and (b) conjunctival to scleral i.e., full segmentation scans for each eye, before image processing as previously described (27). Essentially, motion artefacts were first removed using Fiji-J (NIH, Bethesda, MD) with a Fast Fourier Transform (FFT) bandpass filter (tolerance of direction 90%). Next, the images were processed with MATLAB (The Mathworks, Inc., Natick, Massachusetts, USA) to segment vessels by removing the speckle noise using a median filter and Gaussian smoothing, then applying Frangi filter to enhance vessel features (Figure 2). Finally, local adaptive thresholding was used to binarize the images. The binary images were used to calculate the vessel densities (equation label) of corneal vessels within each sector. Vessel density is defined as the segmented vessels (in white pixel) divided by the sector area (total pixels) i.e., Vessel density = 100 * *P*/*A*; where *P* = 1 for white pixels representing blood vessels, *P* = 0 for black pixels representing the background, and A being the sector area. As a higher signal strength improves the reliability of measurement and allows for better reproducibility (28), we compared the sector with the highest OCTA vessel density with the control sectors. For consistent comparison, inferior and temporal sectors were used as controls for superior-nasal implants. Likewise superior and temporal sectors were used as controls for inferior-nasal implants, and superior and nasal sectors for inferior-temporal implants. These same sectors were kept consistent between visits. Thus, for each Hydrus Microstent sector we have 2 opposing sectors as controls. We confirmed that vessels derived from AS-OCTA images corresponded to episcleral outflow veins, we injected trypan blue (VisionBlue^®^) intra-operatively into the anterior chamber after Hydrus Microstent implantation to highlight episcleral venous vessels. Corresponding AS-OCTA images at 1 month were selected from sectors with and without the implant, and compared to the intra-operative images with highlighted vessels that were manually segmented and overlaid with ImageJ (Figure 3).

### Statistical Analysis

**Figure 4:**
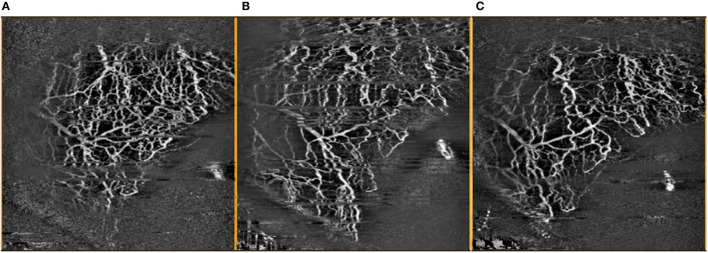
Comparison of OCTA images of inferior temporal episcleral vessels taken **(A)** pre-operatively, **(B)** 1 month post-operatively, and **(C)** 3 months post-operatively, with artefacts removed.

Vessel densities obtained from control sectors without implants were compared to vessel densities from sectors with the Hydrus Microstent in the same eye, with serial comparison analysis performed over the follow-up period (Figure 4). Statistical analysis was performed using Statistical Program for Social Sciences version 27.0.1.0 for MacOS© (2020 SPSS© Inc. IBM Corp, USA). Percentage differences in vessel densities were evaluated using Friedman Test (serial measurements over follow-up) and Wilcoxon Signed Rank Test (paired, compared to baseline). All data were expressed as mean ± standard deviation (SD) when applicable, and *P* < 0.05 were considered statistically significant.

## Results

In this pilot study, we included 25 eyes from 23 subjects undergoing Hydrus Microstent implant surgeries. Patients' mean age was 70.3 ± 1.5 years, with 74% Chinese from our predominantly Asian population, while 61% were female. The mean pre-operative intraocular pressure (IOP) was 15.5 ± 4.0 mmHg, with eyes being on an average of 1.2 glaucoma medications pre-operation, and mean HVF MD was −4.9 ± 3.2 dB. We observed a reduction in mean post-operative IOP at 1 week (11.6 ± 3.1 mmHg, *P* = 0.001), 1 month (12.8 ± 3.1 mmHg, *P* = 0.002), 3 months (12.3 ± 3.2 mmHg, *P* = 0.001), and 6 months (12.5 ± 3.1 mmHg, *P* = 0.002)—none required IOP lowering medications post-operatively during follow-up. There were no significant post-operative complications such as hyphaemia, infection or progression of glaucoma; while no eyes required a repeat surgical procedure during the follow-up period.

All eyes had intra-operative identification of episcleral vessels after Hydrus Microstent implantation to confirm drainage of aqueous humour and intra-operative images were compared with corresponding AS-OCTA images at post-operative day 1 (Figure 3). At baseline, implant sectors have higher vessel densities compared to control sectors for both episcleral images (*p* = 0.03, mean difference of 2.12, 95% CI [0.13, 4.11]) and full-thickness scans (*p* = 0.03, mean difference of 2.21, 95% CI [0.19, 4.23]). We analysed the month 1 AS-OCTA images that clearly corresponded with trypan blue labelled vessels (6 sectors) and observed a reduction in VD of trypan blue labelled vessels comparing sectors with the implant vs. control sectors with no implant (mean difference 1.35 ± 0.5 vs. 0.64 ± 0.2 respectively, *p* = 0.008). We also found that sectors with the Hydrus Microstent implanted showed significant reductions in episcleral vessel density over 6 months (*p* = 0.011), with specific reductions in VD observed at month 1 (*p* = 0.001, mean difference of −3.2, 95% CI [−1.14, −5.10]), month 3 (*p* = 0.004, mean difference of −2.94, 95% CI [−1.12, −4.76]) and month 6 (*p* = 0.039, mean difference of −2.19, 95% CI [−0.29, −4.08]) compared to pre-operative baseline. Meanwhile, control sectors remained unchanged at all time points compared to pre-operative vessel densities (Table 1).

**Table 1:** Optical coherence tomography angiography vessel density measurements of episcleral vasculature in sectors with Hydrus Microstent implants and controls comparing pre-operative and post-operative week 1 and month 1, 3, and 6.

**Assessment time-points**	**Vessel density Mean (±SD)**	* ***P*** **-value**
**Control**		
Pre-operative (*n =* 25)	23.2 (4.3)	0.910**
Week 1 (*n =* 25)	23.0 (3.4)	0.946
Month 1 (*n =* 23)	23.3 (3.5)	0.976
Month 3 (*n =* 16)	22.8 (1.4)	0.538
Month 6 (*n =* 13)	24.0 (3.3)	0.917
**Control**		
Pre-operative (*n =* 24)	24.0 (4.4)	0.723**
Week 1 (*n =* 25)	23.0 (3.4)	0.241
Month 1 (*n =* 23)	23.3 (3.5)	0.114
Month 3 (*n =* 16)	23.2 (4.7)	0.836
Month 6 (*n =* 13)	23.0 (5.1)	0.552
**Hydrus Microstent sector**		
Pre-operative (*n =* 25)	25.6 (3.4)	0.011**
Week 1 (*n =* 25)	24.7 (3.9)	0.326
Month 1 (*n =* 23)	22.5 (3.4)	0.001
Month 3 (*n =* 16)	22.7 (2.8)	0.004
Month 6 (*n =* 13)	23.5 (3.1)	0.039

* *Wilcoxon signed rank test (paired) comparing baseline VD to follow-up VD measurements*.

** *Friedman test for serial VD measurements over follow-up period (n = 25 eyes at baseline, 1 week and 1 month; n = 16 eyes beyond month 3)*.

Similar analysis for full segmentation of AS-OCTA scans i.e., conjunctival, episcleral and scleral layers showed a similar trend i.e., Hydrus Microstent sectors showed significant reduction in VD month 1 (*p* = 0.001, mean difference of −3.27, 95% CI −1.12, −5.42), month 3 (*p* = 0.005, mean difference of −3.24, 95% CI [−1.33, −5.15]) and month 6 (*p* = 0.046, mean difference of −2.18, 95% CI [−0.12, −4.25]) compared to baseline, while control sectors remained unchanged at all time points (Table 2). Of note, these differences were not significant at week 1 in both episcleral (*p* = 0.326, 95% CI −1.09, 3.07) and full segmentation scans (*p* = 0.510, 95% CI −1.45, 2.99).

**Table 2:** Optical coherence tomography angiography vessel density measurements of overall vasculature (conjunctival, episcleral, scleral) in sectors with Hydrus Microstent implants and controls comparing pre-operative and post-operative week 1 and month 1, 3, and 6.

**Assessment time-points**	**Vessel density Mean (±SD)**	* ***P*** **value (compared to pre-operative)**
**Control**		
Pre-operative (*n =* 25)	22.9 (4.4)	0.699**
Week 1 (*n =* 25)	22.8 (3.2)	0.757
Month 1 (*n =* 23)	23.1 (4.0)	0.761
Month 3 (*n =* 16)	23.1 (2.1)	0.717
Month 6 (*n =* 13)	24.4 (3.0)	0.701
**Control**		
Pre-operative (*n =* 24)	24.2 (4.3)	0.536**
Week 1 (*n =* 25)	23.2 (4.2)	0.153
Month 1 (*n =* 23)	23.2 (3.0)	0.153
Month 3 (*n =* 16)	23.3 (4.7)	0.301
Month 6 (*n =* 13)	24.0 (4.5)	0.601
**Hydrus Microstent sector**		
Pre-operative (*n =* 25)	25.7 (3.6)	0.021**
Week 1 (*n =* 25)	25.0 (4.2)	0.510
Month 1 (*n =* 23)	22.5 (3.8)	0.001
Month 3 (*n =* 16)	22.5 (2.9)	0.005
Month 6 (*n =* 13)	23.6 (3.5)	0.046

* *Wilcoxon signed rank test (paired) comparing baseline VD to follow-up VD measurements*.

** *Friedman test for serial VD measurements over follow-up period (n = 25 eyes at baseline, 1 week and 1 month; n = 16 eyes beyond month 3)*.

## Discussion

In this pilot study, AS-OCTA detected a post-operative reduction in episcleral vessel density in sectors with the Hydrus Microstent implant compared to control sectors without implants. We correlated these vessels with intraoperative imaging that highlighted the aqueous outflow tracts using trypan blue. Our observations may seem counterintuitive since trabecular bypass MIGS devices such as the Hydrus Microstent are meant to enhance flow to collector channels (29). We postulate that the apparent reduction in AS-OCTA derived vessel density measurements may be attributed to increased aqueous humour flow in the episcleral veins, thereby reducing the signal intensity or phase differences detected by the AS-OCTA (30). Changes in vessel density in the control sectors could have been a result of cataract surgery itself, which may also increase aqueous flow to a lesser extent (31, 32). It is also possible that the Hydrus Microstent leads to changes in aqueous outflow in a differential manner, i.e., some vessels with greater aqueous flow, while others with decreased flow—leading to an overall AS-OCTA detection of decreased vessel density (33).

The reduction in vessel densities could also be due to the cessation of IOP-lowering medications, many of which are associated with hyperaemia (34). To control for potential confounders, we compared Hydrus Microstent sectors with control quadrants without the implant within the same eye, such that all quadrants were subjected to potential effects of medications and cataract surgery. However, we do recognise this study's limitations and cannot exclude any local quadrant effects of prostaglandin use if applicable (35). Lastly, vessel densities were found to be higher in sectors with Hydrus Microstent compared to control sectors at baseline. One explanation could be that Hydrus Microstent sectors included more inferior-nasal sectors, which were associated with the highest vessel volume of aqueous outflow channels out of all ocular sectors, and hence greater vessel densities (36, 37). Nonetheless, we recognise that AS-OCTA cannot directly detect aqueous outflow, but instead measures episcleral vessel density as a potential surrogate (38). As such, current AS-OCTA technology might not yet be an ideal imaging modality for the detection of conventional aqueous outflow. However, our study was a unique opportunity to confirm the location of the outflow tracts using trypan blue, and allowed us to compare pre- and post-procedure changes in vessel density in the sectors with the implant, vs. control sectors without any implant. Further validation studies using larger sampling sizes and alternative methods for aqueous outflow and episcleral vessel delineation, such as aqueous angiography, are needed to confirm our observations.

Currently, imaging the aqueous outflow tracts are not performed in the clinical setting due to the need to inject a contrast agent into the anterior chamber. Thus, AS-OCTA could provide a non-invasive alternative to imaging the episcleral venous plexus as an adjunctive or surrogate for evaluating the aqueous outflow (39). The AS-OCTA has been previously shown to detect increased episcleral vessel density due to increased episcleral venous flow leading to raised intraocular pressure (40), or reduction in episcleral vessel density following anterior segment ischemia (41). Although a previous report suggested that AS-OCT (non-angiographic) imaging did not detect changes in aqueous outflow after successful trabecular-targeted MIGS (120-degree trabectome or 360-degree suture trabeculotomy) during 3 month follow-up (42), our study specifically examined AS-OCT angiography to delineate the episcleral venous plexus following trabecular bypass MIGS. As AS-OCTA imaging is rapid and non-contact, repeated serial post-operative scans can be taken for comparison (43), and may be useful for pre-operative assessment to guide trabecular bypass device placement.

Though MIGS has been viewed as a safe surgical option for lowering IOP in carefully selected eyes, variable efficacy has been attributed to non-optimal surgical placement (44). Traditionally MIGS devices are placed in the nasal angle (45), but pre-operative imaging assessment using modalities such as AS-OCTA may be able to optimise surgical planning and the location of device implantation. Past studies have demonstrated the non-uniform nature of AHO around the limbus, which may vary over time and differ between eyes (46). Hence it is unclear how these variations might affect the surgical outcomes and decision on the most optimal location for trabecular bypass MIGS. Nonetheless, this highlights the unmet need for an imaging modality to allow for preoperative assessment to individualise MIGS implantation for patients.

Despite the promising observations from our pilot study, we would like to highlight several challenges with using the AS-OCTA to image the episcleral venous plexus. Firstly, scan techniques will have to be adjusted for the anterior segment as these systems were originally designed for the posterior segment, hence anterior segment images are motion-sensitive and regions of interest are more difficult to match in serial scans (21). In our study, we recognise the limitations of manually segmenting the episcleral layers from AS-OCTA imaging to minimise effects from conjunctival vessels on our analysis, and thus correlated our images with intraoperative dye labelled vessels and observed reduction in overall vessel density from fully segmented scans as well. Secondly, AS-OCTA derived vessel density measurements may be underestimated due to limited detection of smaller vessels (47), or overestimated due to projection and motion artefacts (48). Thus, repeated serial measurements were performed to reduce random errors and confirm our observations. Thirdly, the AS-OCTA does not directly image the aqueous outflow tracts such as the Schlemm's canal and collector channels (49). Ideally we should perform aqueous angiography to assess the actual flow through the collector channels—thus the Hydrus implant may not increase the flow in the collector channels. However, we used the AS-OCTA imaging of the episcleral venous plexuses as a surrogate *in vivo*, as flow may be detected because the vessels are partially filled with both clear aqueous humour and blood; and we had further confirmed increased aqueous outflow using intraoperative imaging with trypan blue. Lastly, although we have excluded patients with advanced PACG, we have included patients with both mild to moderate PACG and primary open-angle glaucoma (POAG). Ideally we would have studies with larger sample sizes that exclude PACG eyes, but as this is a pilot study primarily focused on investigating the feasibility of using AS-OCTA for imaging of episcleral vessels pre- and post-MIGS, we have decided to include PACG eyes to aid in estimation purposes. Despite these limitations, our study suggests that AS-OCTA is a promising non-invasive imaging tool that is readily available in the clinics, that may be useful in assessing changes in episcleral vessel density secondary to trabecular bypass MIGS.

In summary, our pilot study suggests that AS-OCTA detects changes in episcleral vessel density before and after trabecular bypass MIGS implantation in sectors with the implant compared to control sectors. The reduction in episcleral vessel density is observed to occur over 3 to 6 months after surgery, which requires further validation in future studies to examine the potential clinical application of AS-OCTA imaging for this indication.

## Data Availability Statement

The raw data supporting the conclusions of this article will be made available by the authors, without undue reservation.

## Ethics Statement

The studies involving human participants were reviewed and approved by National Healthcare Group Domain Specific Review Board. The patients/participants provided their written informed consent to participate in this study.

## Author Contributions

All authors substantial contributions to conception and design, acquisition of data, or analysis and interpretation of data, drafting the article or revising it critically for important intellectual content, and final approval of the version to be published.

## Funding

This work was supported by Singapore Imaging Eye Network (SIENA), project no. NMRC/CG/C010A/2017_SERI and SERI-NTU Advanced Ocular Engineering (STANCE) Program.

## Conflict of Interest

The authors declare that the research was conducted in the absence of any commercial or financial relationships that could be construed as a potential conflict of interest.

## Publisher's Note

All claims expressed in this article are solely those of the authors and do not necessarily represent those of their affiliated organizations, or those of the publisher, the editors and the reviewers. Any product that may be evaluated in this article, or claim that may be made by its manufacturer, is not guaranteed or endorsed by the publisher.

## Abbreviations

AHO: aqueous humour outflow
AS-OCTA: anterior segment optical coherence tomography angiography
BCVA: best-corrected visual acuity
FFT: Fast Fourier Transform
HVF: Humphrey Visual Field
ICG: indocyanine green
IOP: intraocular pressure
MD: mean deviation
MIGS: minimally invasive glaucoma surgery
OCTA: optical coherence tomography angiography
PACG: primary angle-closure glaucoma
POAG: primary open-angle glaucoma
VD: vessel density.
